# Worth a Second Look: Report of a Rare Traumatic Pisiform Fracture Following a Bicycle Fall

**DOI:** 10.1155/cro/2097620

**Published:** 2026-02-16

**Authors:** Yannic-Tomas Abulesz, Franz Kralinger

**Affiliations:** ^1^ Department for Trauma Surgery, Klinik Ottakring, Vienna Hospital Association, Austria, Vienna; ^2^ PhD Program Musculoskeletal and Dental Research, Medical University of Vienna, Austria, Vienna, meduniwien.ac.at

**Keywords:** carpal bones, case report, pisectomy, pisiform fracture, wrist

## Abstract

Fractures of the pisiform bone are difficult to diagnose due to the often unsuspicious conventional X‐ray images and nonspecific clinical presentation. The following case presents a 29‐year‐old patient who suffered a bicycle fall onto their right hand and experienced prolonged symptoms despite the initial lack of evidence of a fracture. The fractured pisiform bone, which was eventually detected in a CT scan, was successfully treated with a 4‐week period of cast immobilization. The existing conservative and surgical treatment options of a pisiform bone fracture are discussed.

## 1. Introduction

We report the case of a young male with a pisiform fracture obtained through a bicycle fall. The fracture was missed on the first visit to the trauma ward due to unspecific symptoms and unsuspicious X‐ray images but was later detected through more specific clinical findings and a CT scan. This presentation shall draw attention to possible diagnostic pitfalls in carpal fracture diagnosis.

## 2. Case Presentation

**Figure 1:**
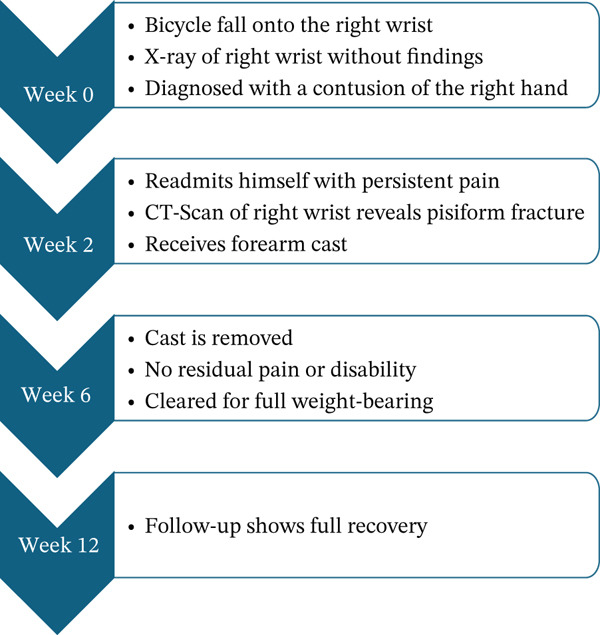
The course of the patient.

Figure 1 shows a graphical overview of the course of the patient.

### 2.1. Patient Information

A 29‐year‐old patient presented to our outpatient clinic due to a fall from a bicycle onto the right wrist and right elbow. The pain was mainly localized in the area around the right wrist, with only a small skin injury reported in the elbow area. Otherwise, there was no pain or complaints.

### 2.2. Clinical Findings

On the right wrist, apart from external soiling of the palm, there was no evidence of injuries such as swelling or hematoma. Tenderness to pressure could only be elicited weakly around the distal ulna; the anatomical snuffbox and the distal radius were not tender to pressure.

The range of motion in the wrist was not restricted in any plane and could be demonstrated painlessly. Distal circulation, motor skills, and sensation were unrestricted.

In the area of the elbow, there was only a small unsoiled laceration wound; there were no functional limitations at the elbow joint.

### 2.3. Diagnostic Assessment

**Figure 2:**
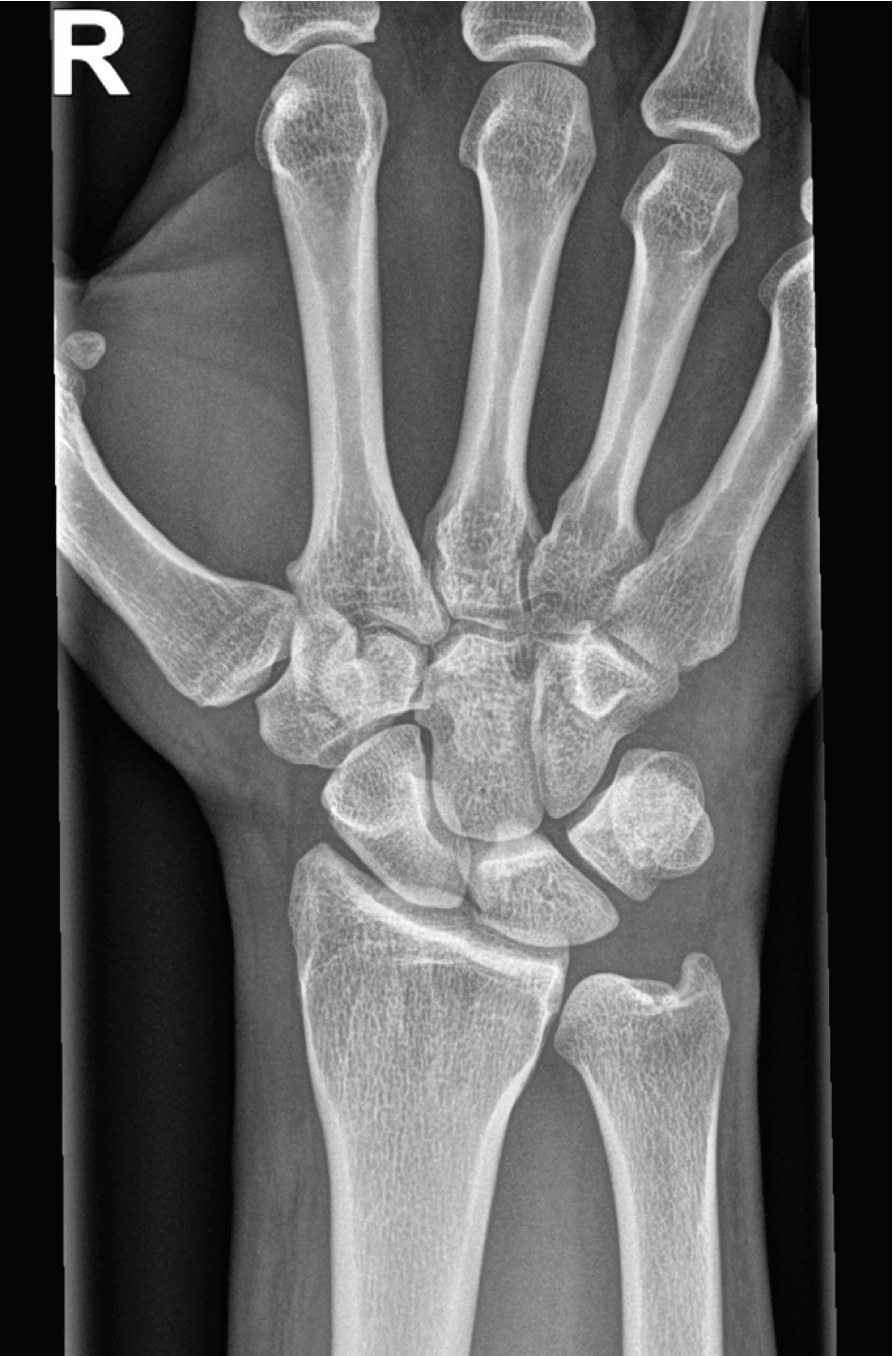
Conventional X‐ray (Week 0); ap view.

**Figure 3:**
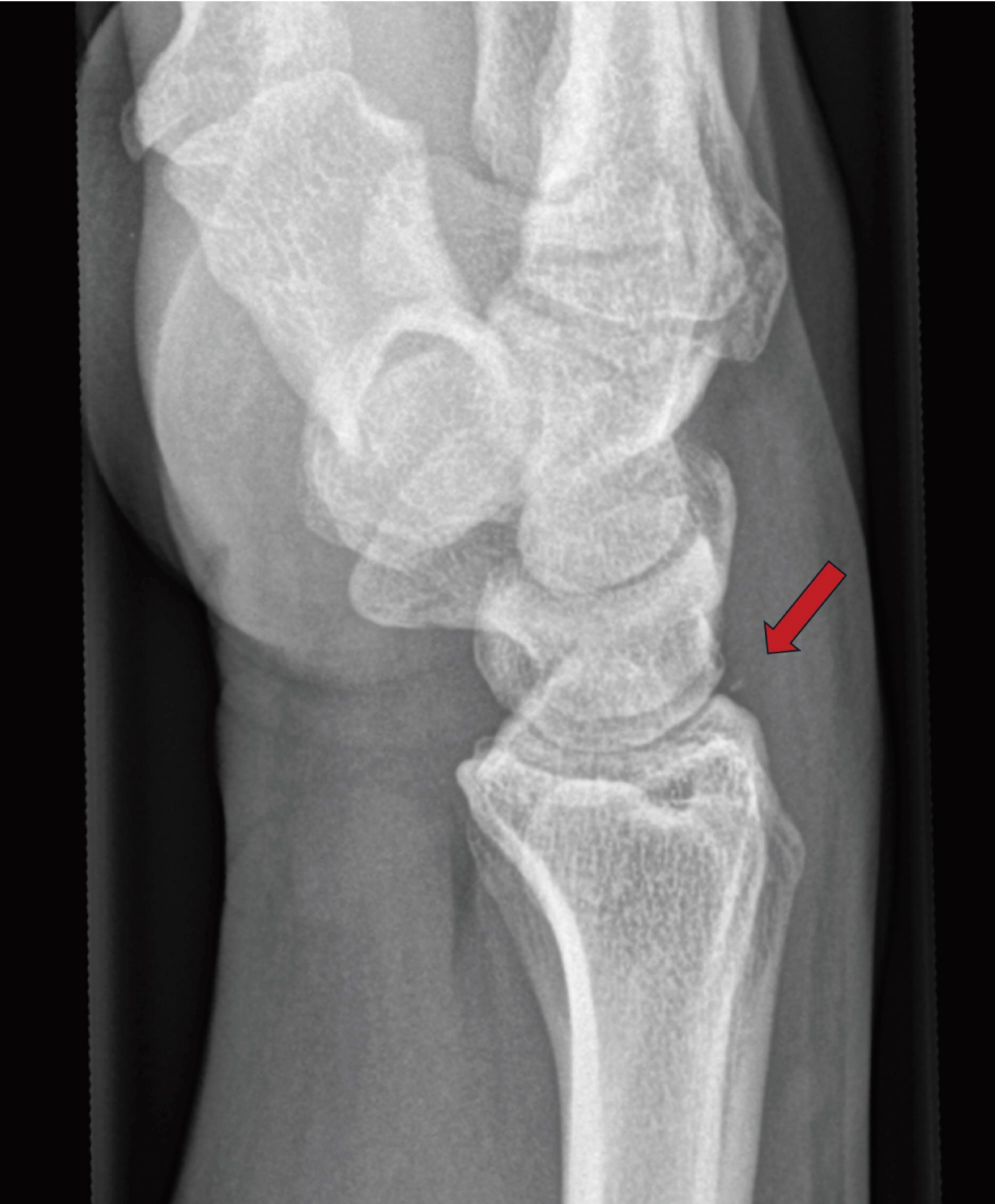
Conventional X‐ray (Week 0); lat. view–small ossicle visible.

A conventional X‐ray of the right wrist in two planes was performed (Figures 2 and 3), which showed no evidence of a recent traumatic bone change. Only a small ossicle over the dorsal side of the wrist was noticeable (marked by a red arrow); however, there was no clinical presentation such as tenderness to pressure or movement restrictions in that area.

Due to the mild symptoms and unremarkable conventional X‐ray, the patient was discharged with a pain‐relief ointment bandage, and a contusion of the right wrist was recorded as the diagnosis.

The wound on the elbow joint was treated and healed without complications.

After 2 weeks, the patient presented again and reported persistent pain around the ulnar carpus. A repeat clinical examination with palpation of all bony landmarks was performed, which again did not reveal a clearly localizable point of tenderness. According to the patient, the pain occurred mainly when performing ulnar deviation in combination with flexion. The pain was then localizable along the flexor carpi ulnaris (FCU) tendon.

Upon re‐examination of the X‐ray images, with a focus on the ulnar carpus, a minimal irregularity around the hamate bone and pisiform bone was noted; therefore, a CT scan of the carpus was ordered (exemplary Figure 4a,b—fracture marked by red arrows).

Figure 4(a, b) CT scan of the carpus (Week 2) in coronal and axial plane–visible pisiform fracture.

**Figure:**
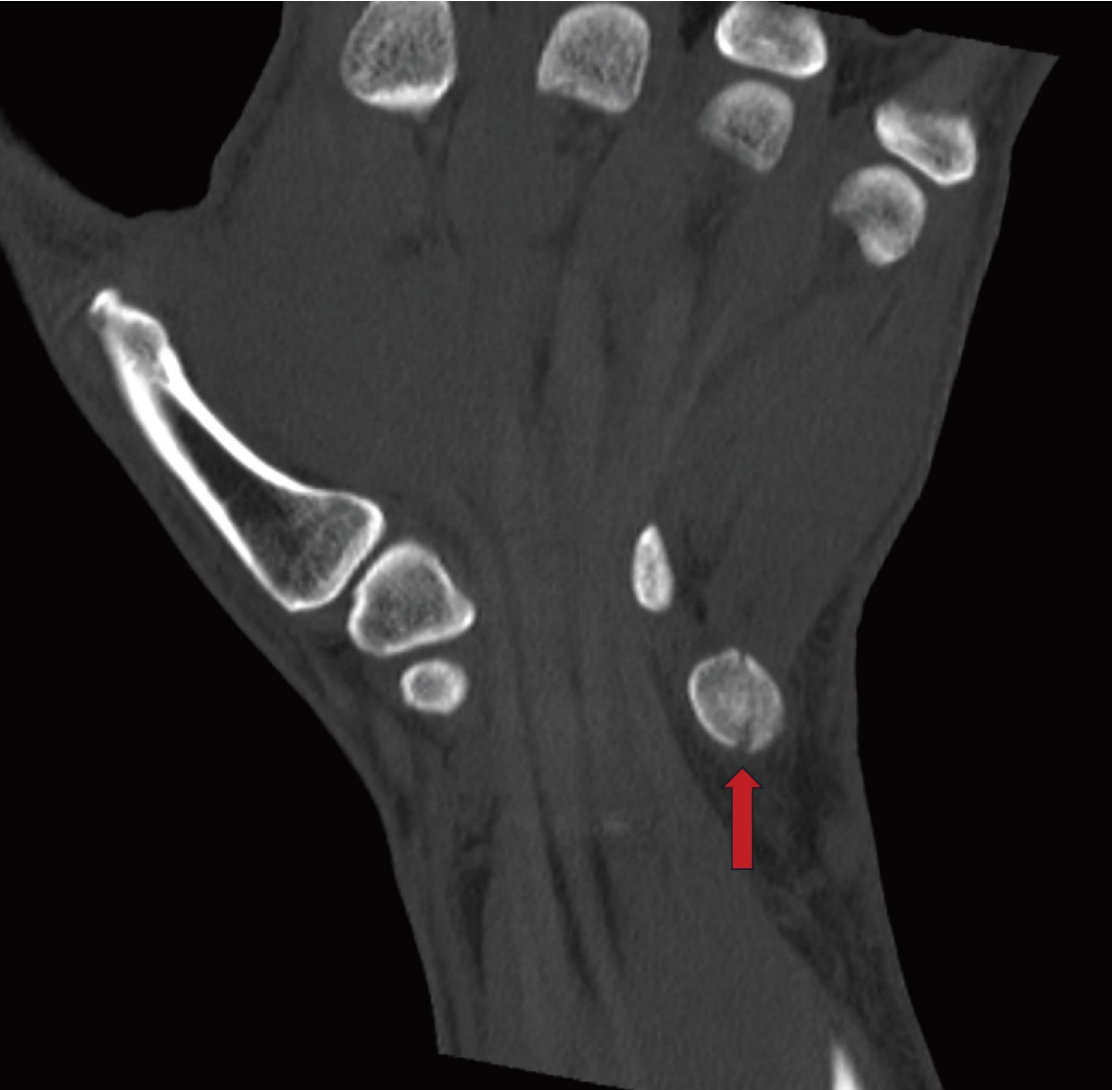
(a)

**Figure:**
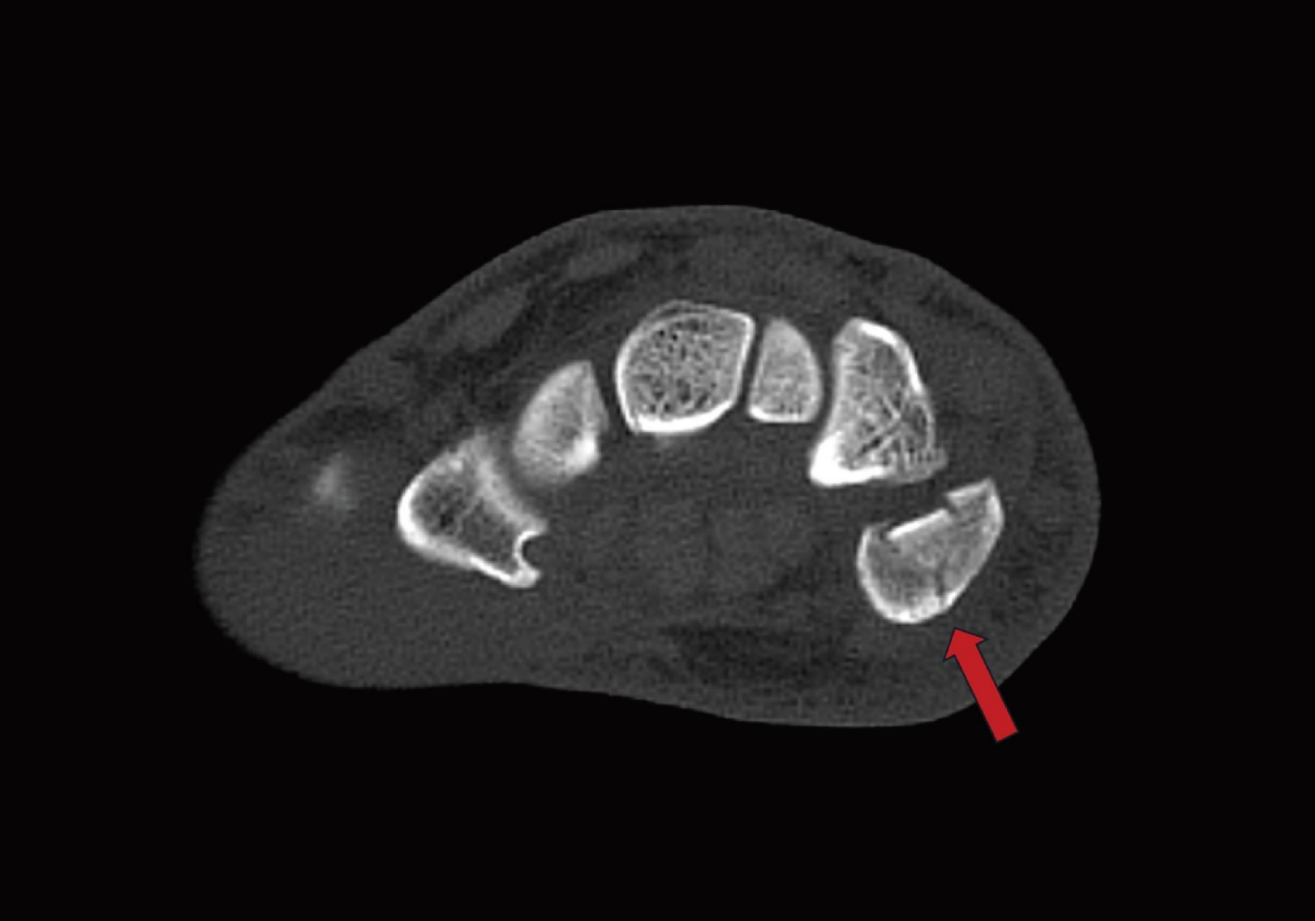
(b)

The CT scan ultimately revealed an isolated, nondisplaced fracture of the pisiform bone.

### 2.4. Treatment

Due to the absence of pathological findings in motor skills, circulation, and sensation, specifically a negative Froment sign, conservative treatment was initiated and the patient was immobilized in a forearm cast.

### 2.5. Outcome

**Figure 5:**
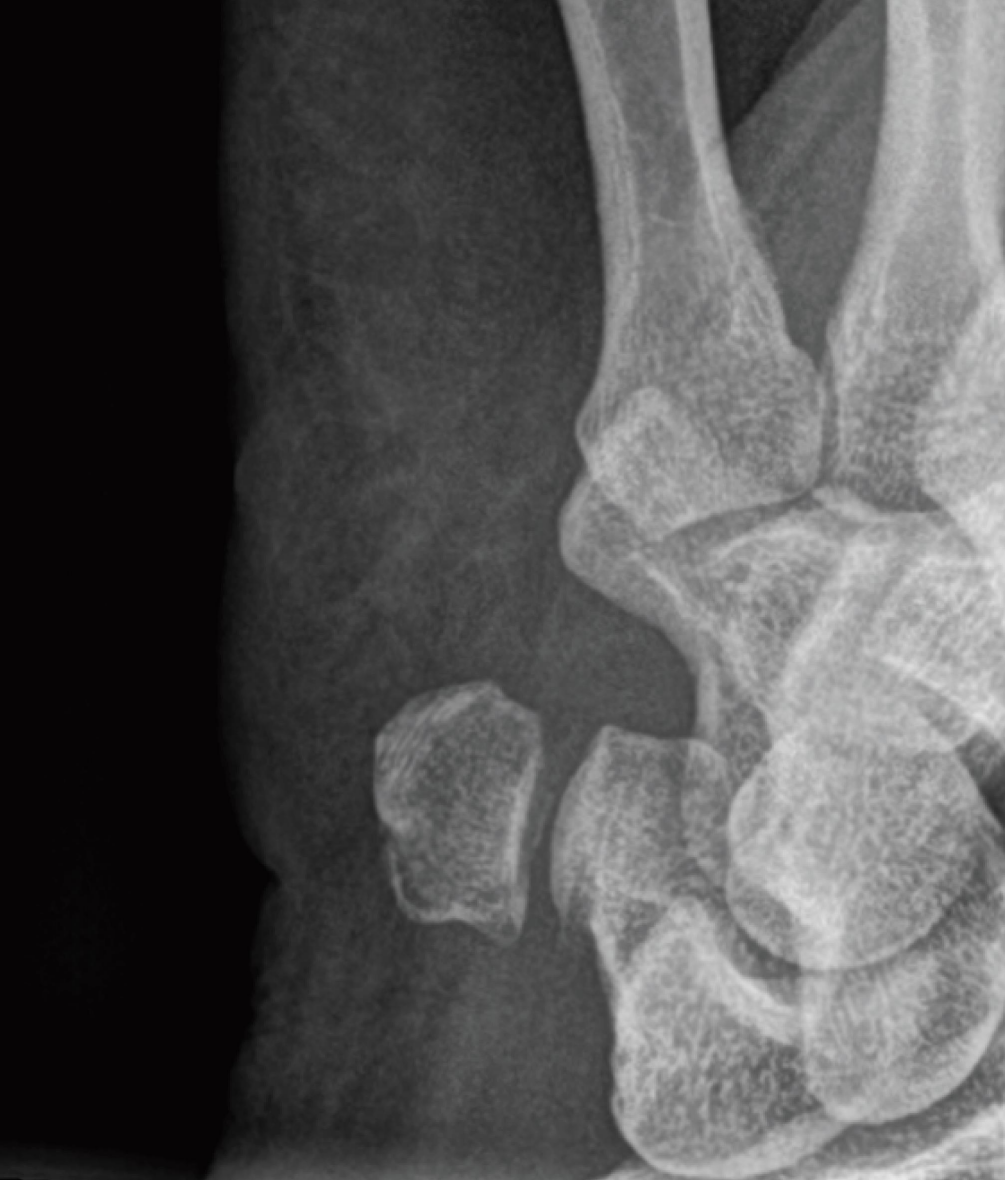
Conventional X‐ray after 4 weeks of cast immobilization (Week 6); pisiform view.

The further course of conservative treatment was uneventful, and after 4 weeks of continuous immobilization, the cast could be removed without remaining pain or secondary dislocation on conventional radiographic pisiform views (Figure 5), and the patient was cleared for full weight‐bearing. A follow‐up after 3 months showed no irregular findings; the patient was pleased with the outcome of conservative fracture treatment.

## 3. Discussion

### 3.1. Available Literature

The pisiform bone is a pea‐shaped bone that articulates posteriorly with the triquetrum bone and attaches anteriorly to the FCU tendon and the transverse carpal ligament [1]. Isolated fractures of the pisiform bone are extremely rare, accounting for only 1%–2% of all carpal bone fractures, which themselves are rare [2]. They occur from direct impact trauma to the hypothenar eminence or as avulsion fractures from forced hyperextension of the wrist or a combination of both mechanisms [2, 3]. Fatigue fractures have only been described in isolated cases [4].

Clinically, the fracture manifests as perifocal pain around the pisiform bone, which can radiate into the hypothenar region or the FCU tendon. Because the pisiform bone forms the ulnar wall of Guyon′s canal, sensory deficits in the distal supply areas can also occur due to its proximity to the ulnar nerve [5].

In regular wrist X‐rays, fractures of the pisiform bone are often difficult or impossible to detect; an additional pisiform view (45° reverse oblique) and a carpal tunnel view are recommended [6], and in doubtful cases, a CT scan of the carpus is necessary.

Due to the rarity of the fracture, there is no evidence‐based treatment guidelines. Nondisplaced fractures are treated conservatively with plaster‐cast immobilization of the wrist [2, 5, 7] or a wrist splint [8] for 4–6 weeks in the existing literature. In a recent publication, a pisiform fracture has been treated with an ulnar gutter splint in slight palmar flexion for only 2 weeks with equally satisfying results [9]. Although a decreased timespan of immobilization is desirable, whether this comes at the cost of an increased risk of dislocation or nonunion is unclear.

In the case of significant fracture displacement with dysfunction of the FCU tendon or a comminuted fracture, excision of the pisiform bone can achieve good results without limitation of range of motion and with reliable pain reduction; this procedure can also be performed later in case of symptomatic nonunion [10] or posttraumatic pisotriquetral arthritis [11]. Internal fixation has no relevance in pisiform fractures [1]. Any nerve damage to the ulnar nerve is usually considered as neurapraxia, and the symptoms should improve within 6 weeks; if this does not happen, nerve exploration is indicated [4]. Other authors recommend immediate excision of the pisiform bone and additional decompression of Guyon′s canal in the presence of sensory disturbance in the supply area in combination with a displaced fracture [12, 13].

### 3.2. Takeaway‐Lessons

Pisiform fractures result from palmar impact trauma to the dorsally extended hand. In cases of clinical suspicion, such as tenderness above the pisiform bone, specialized radiographs should be obtained, or a CT scan of the wrist should be performed.

Conservative management via 4 weeks of cast immobilization is indicated for nondisplaced and minimally displaced pisiform fractures. In the event of major dislocation or complications such as pain or persistent sensory disturbances after cast immobilization, excision of the pisiform bone may become necessary.

## Funding

This study was supported by Medizinische Universitat Wien/KEMÖ, which provided open access funding.

## Ethics Statement

The patient has given informed consent to the publication of this case and the corresponding radiographic images, a signed consent form rests with the authors. A formal ethics review has been waived by the responsible ethics committee. This case report has been drafted in accordance with CARE guidelines.

## Conflicts of Interest

The authors declare no conflicts of interest.

## Data Availability

The data that support the findings of this study are available on request from the corresponding author. The data are not publicly available due to privacy or ethical restrictions.
